# Region‐of‐interest intra‐arc MV imaging to facilitate sub‐mm positional accuracy with minimal imaging dose during treatment deliveries of small cranial lesions

**DOI:** 10.1002/acm2.13769

**Published:** 2022-09-02

**Authors:** Cody Church, David Parsons, Alasdair Syme

**Affiliations:** ^1^ Department of Physics and Atmospheric Science Dalhousie University Halifax Nova Scotia Canada; ^2^ Department of Radiation Oncology University of Texas Southwestern Medical Center Dallas Texas USA; ^3^ Department of Radiation Oncology Dalhousie University Halifax Nova Scotia Canada

**Keywords:** image dose reduction, MV imaging, sub‐mm positioning

## Abstract

**Purpose:**

To automate the generation of region‐of‐interest (ROI) apertures for use with megavoltage imaging for online positional corrections during cranial stereotactic radiosurgery.

**Materials and methods:**

Digitally reconstructed radiographs (DRRs) were created for a 3D‐printed skull phantom at 5 degree gantry angle increments for a three‐arc beam arrangement. At each angle, 3000 random rectangular apertures were generated, and 100 shifts on a grid were applied to the anatomy within the frame. For all shifts, the mutual information (MI) between the shifted and unshifted DRR was calculated to derive an average MI gradient. The top 10% of apertures that minimized registration errors were overlaid and discretely thresholded to generate imaging plans. Imaging was acquired with the skull while implementing simulated patient motion on a linac. Control point‐specific couch motions were derived to align the skull to its planned positioning.

**Results:**

Apertures with a range of repositioning errors less than 0.1 mm possessed a 42% larger average MI gradient when compared with apertures with a range greater than 1 mm. Dose calculations with Monte Carlo exhibited an 84% reduction in the dose received by 50% of the skull with the 50% thresholded plan when compared to a constant 22 × 22 cm^2^ imaging plan. For all different imaging plans (with and without motion), the calculated median 3D‐errors with respect to the tracking of a metal‐BB fiducial positioned at isocenter in the skull were sub‐mm except for the 80% thresholded plan.

**Conclusions:**

Sub‐mm positional errors are achievable with couch motions derived from control point–specific ROI imaging. Smaller apertures that conform to an anatomical ROI can be utilized to minimize the imaging dose incurred at the expense of larger errors.

## 1 INTRODUCTION

Cranial stereotactic radiosurgery (SRS) has been shown to achieve high tumor control rates (greater than 95%) in pituitary adenomas,^1^ high obliteration rates (78%) of arteriovenous malformations,^2^ and lengthen the median survival of patients with 1–3 brain metastases while subsequently reducing neurocognitive decline.^3^ This precision therapy delivers large doses in a single fraction (or a small number of fractions in the case of stereotactic radiotherapy) and uses techniques that promote rapid dose falloff of dose outside of the targets. In the case of certain functional disorders (e.g., trigeminal neuralgia), prescription doses can be as high as 90 Gy^4^ and beam‐on‐times can be as long as 19.4 ± 0.6 min with an average delivered MU of 19 444 ± 611 at 1000 MU/min.^5^ Trends in SRS treatment have moved away from invasive headframes toward noninvasive thermoplastic mask‐based immobilization. Studies have shown that the combination of long treatment times and mask‐based immobilization can lead to patient motions on the order of 2–3 mm.^6^, ^7^ The dosimetric consequences of intrafraction motion on small targets and the surrounding tissues have been previously reported,^8^ and those findings suggest that additional methods, beyond frameless immobilization, could be beneficial for ensuring accurate treatment delivery.

Various considerations go into the choice of the size of margins placed around the gross tumor volume, one of which being the expected patient motion during therapy. However, increasing numbers of centers have been reporting the use of no planning target volume margins (49.1% of centers).^9^ Although techniques for patient alignment vary across centers, the majority utilize magnetic resonance imaging fusion with simulated computed tomography (CT) for planning and cone beam CT (CBCT) or registration with digitally reconstructed radiographs (DRRs) from volumetric CTs on the day for the verification of isocenter position with respect to the patients’ coordinate frame.^10^, ^11^, ^12^ Beyond high‐precision setup, intrafractional motion management strategies have made their way into various treatment modalities, such as Gamma Knife (Elekta AB, Crawley, United Kingdom),^6^ which utilizes infrared‐based monitoring or optical surface monitoring, or CyberKnife (Accuray Inc., Sunnyvale, CA),^13^, ^14^ which monitors motion with periodic orthogonal kilovoltage (kV) imaging every 5–150 s (user‐defined). Several intrafraction motion monitoring systems for C‐arm linear accelerators (linacs) are currently clinically available, some of which are (1) the ExacTrac system (Brainlab AG, Munich, Germany), which can utilize an infrared camera monitoring device for fiducial‐tracking in conjunction with two floor‐mounted kV X‐ray systems to 3D‐localize the patient by comparing images to plan‐generated DRRs.^15^ This system requires hardware external to the accelerator and is not continuous; (2) optical imaging with lasers or speckled light patterns, such as the AlignRT system (Vision RT, London, UK), which relies on skin‐monitoring and has several limitations: It can lead to false positives and false negatives, it requires less restrictive immobilization masks that could lead to motion, and imaging could be occluded by the onboard imaging arms of the gantry.^16^, ^17^ For C‐arm linacs that are not equipped with an imaging system like ExacTrac, the development of novel motion minimization schemes would be beneficial. In this work, we explore the use of intra‐arc imaging with the megavoltage (MV) imaging beam to correct for motions detected in the beams‐eye‐view (BEV).

The imaging dose accrued with MV imaging^18^ or CBCT^19^ has been a concern with image‐guided radiation therapy, in particular with pediatric cases. Several works have explored the use of region‐of‐interest (ROI) CBCT^20^, ^21^ and MV‐CT^22^ and found possible dose reductions of 16%–90% and 15%–75% with ROI‐based CBCT and MV‐CT, respectively, when compared with full‐field imaging. While cupping artifacts were present, using smaller imaging apertures with the MV‐imaging beam presented minimal losses to contrast‐to‐noise ratio (CNR) when delineating bony anatomy.^22^ We propose that BEV optimal imaging apertures can be used to achieve sub‐mm intra‐arc target localization by deriving necessary couch‐shifts on a control point–specific basis with image registration. The frequency of imaging could be user‐defined to balance tolerance for expected motion with imaging dose and treatment delivery efficiency. Herein we report a method for the generation and evaluation of apertures for ROI imaging and quantify the results of image registration tests as a function of aperture size. The technique is then used to demonstrate the feasibility of correcting for motions owed to mechanical imperfections during gantry rotation, as well as intrafractional motion with the MV imaging beam in a clinical setting.

## 2 MATERIALS AND METHODS

### 2.1 Phantom fabrication

**FIGURE 1:**
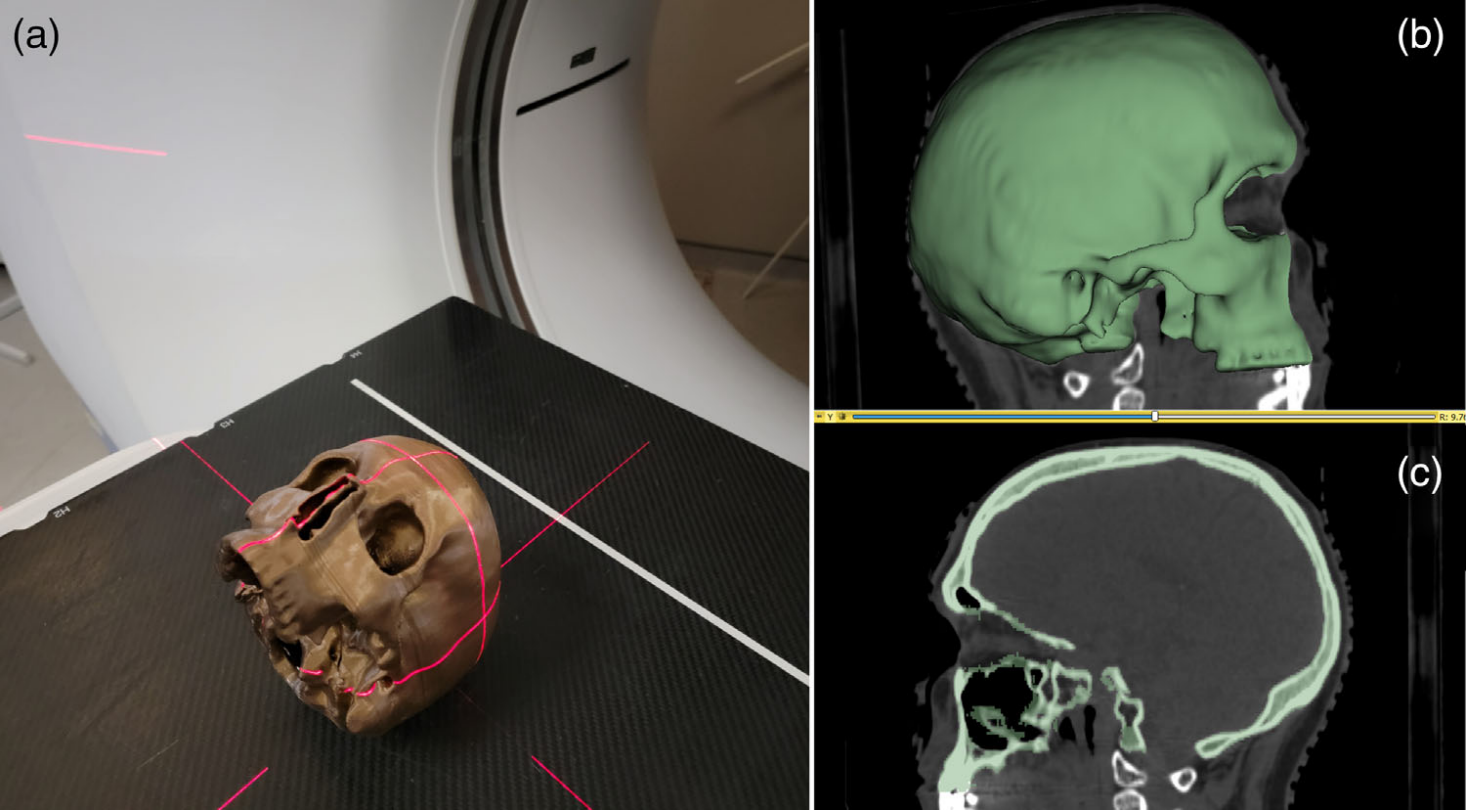
(a) The printed skull aligned on the computed tomography (CT)‐bed for imaging. (b) The 3D model of the skull embedded in a sagittal view of the skull. (c) A sagittal view of the skull with the contoured regions highlighted in green

The skull of an anonymized cranial SRS case at Nova Scotia Health Authority was contoured (with the exclusion of the mandible) with 3D Slicer (https://www.slicer.org/), as shown in Figure 1. The skull was 3D printed at 90% scale with a copper‐doped PLA filament (3D Printing Canada, Hamilton, Canada) using a 0.3 mm layer height and at a print speed of 40 mm/s. The infill factor for the skull was chosen to be 100% as it maximized the Hounsfield units (HU) when scanned with a CT‐scanner (396.53 ± 20.19). The purpose of this choice was to fall within the range of reported HU for real cranial bone that contains a composite of various bone types (cancellous, cortical), which produce HU within the range of 400–1500. To emulate brain tissue, the skull was filled with gelatin. A 2 mm metal‐BB was placed at the center of the skull after filling the skull halfway and letting the gelatin cure for 3 h. The BB served as a tracking fiducial during imaging and was placed at isocenter. The rest of the skull was then filled and sealed, without filling the sinus–cavities with gelatin. The back of the skull was planned and leveled to ensure a consistent placement (pitch‐independent) between the CT‐couch and the couch on the linac.

### 2.2 Treatment workflow

**FIGURE 2:**
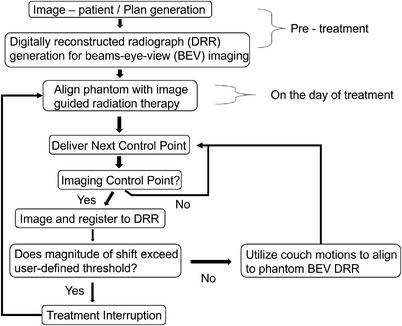
An example of a clinical workflow for control point–specific image repositioning

A method for control point–specific patient position correction is shown in Figure 2. The generic workflow allows for an arbitrary number of gantry and couch angle combinations for imaging, defined as imaging points, distributed according to user preference. For the purposes of this evaluation, imaging points were evaluated at all points throughout a series of noncoplanar arc geometries as described later (to within a 5 degree gantry angle resolution).

To begin, a high‐resolution CT scan (0.625 mm slice thickness) was acquired of the phantom and imported into the Eclipse treatment planning system (version 15.3; Varian Medical Systems, Inc. Palo Atlo, CA). A treatment plan consisting of three arcs was created for the purpose of defining the treatment and imaging geometry (in particular, the location of isocenter that is essential for the subsequent steps of the algorithm). The arc geometry in this study consisted of one full axial arc and two partial arcs with the couch rotated to ±45 degrees. For the imaging‐arc with the couch angle at 0 degrees, the electronic portal imaging device (EPID) was set to 50.0 cm in the vertical direction. When the couch was at ±45 degrees, the EPID was extended to 80.0 cm in the vertical direction in order to avoid couch‐EPID collisions. The gantry–couch angle combinations in this work allowed for imaging at every control point. However, there are gantry–couch angle combinations that would preclude the possibility of BEV imaging due to collisions of the EPID with the couch and/or patient. For example, with a vertex arc (gantry ranging from 180.0 to 15 degrees in 5 degree increments, Varian IEC coordinates), nearly half of the imaging control points could be inaccessible (approximately from 250 to 345 degrees Varian IEC coordinates) due to collisions of the EPID with the couch or patient. The full specifications of the arcs are shown in Table 1. Control points, which are defined as any unique gantry and couch angle combination, were defined every 5 degrees of gantry rotation. Isocenter was located at the center of the metal‐BB. The DICOM plan object and images were then exported for further use in MATLAB (R2020b, The MathWorks, Inc. Natick, MA).

**TABLE 1:** Arc geometry specifications in Eclipse coordinates

Arc	Couch angle (degrees)	Gantry span (degrees)
One	0	180.1–179.9, CW
Two	45	179.9–355, CCW
Three	315	180.1–5, CW

From this volumetric CT, a MATLAB‐generated DRR was created for each control point using Siddon's method.^23^ Prior to DRR generation, the voxels within the volumetric CT in a 60 × 60 × 40 pixel neighborhood around the center of the BB were assigned a value of 36.1 HU; this corresponded to the average HU of the gelatin material within the skull (taken from a 20 × 20 × 20 voxel neighborhood in slices that did not contain the BB). To facilitate efficient image acquisition, imaging plans were created for use in developer mode on a TrueBeam STx platform (Varian Medical Systems Inc. Palo Alto, CA). The plans consisted of the arcs described in Table 1 with imaging control points (where a high‐resolution MV image was acquired) defined every five degrees of gantry rotation. Prior to the delivery of the imaging plans, a CBCT was acquired to align the phantom to its planning CT with errors along each linear couch axis being less than 0.1 mm, and couch rotation (yaw) errors being less than 0.1 degree. This form of alignment will leave residual positional errors that result from the disagreement between the MV and kV isocenters, which are typically found to be less than 1 mm. In this study, image acquisition and analysis were decoupled as the analysis was performed in MATLAB (described later in Section 2.4). In a clinical setting, the workflow would be altered to permit image registration, and repositioning to occur in pseudo real‐time at a given control point.

### 2.3 Control point–specific apertures

**FIGURE 3:**
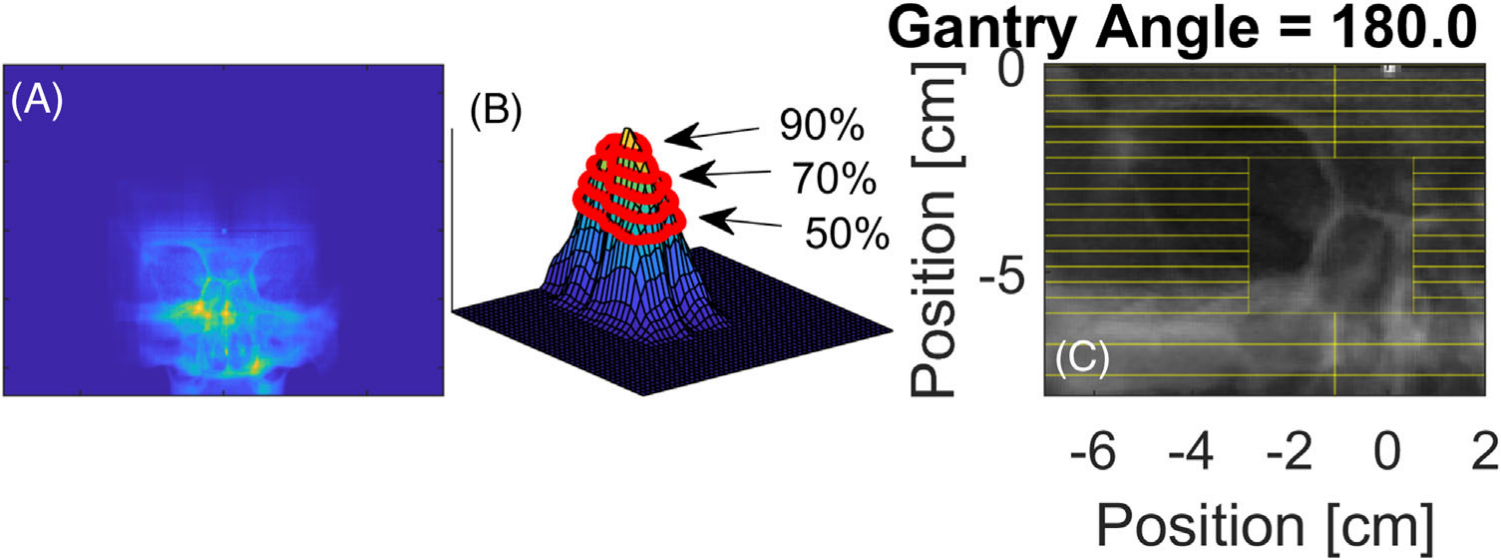
(a) Mask overlay of top 10% of apertures, which minimized registration errors. (b) Topographical map created from sum of top 10% apertures, which minimized registration, peak indicates most common pixels shared by apertures, and contours depict thresholds for aperture designs. (c) An aperture design derived from the 80% threshold level depicted in (b)

One objective of this work is to identify apertures that are capable of providing accurate registration information following motion, regardless of the direction of that motion. Such a set of apertures is required at all possible imaging points along the arcs. A second objective is to determine how small an aperture can be used such that accurate registration information is still generated while minimizing the dose delivered to acquire that information. Apertures that conformed to an anatomical ROI were explored for use in control point–specific repositioning with image registration. To choose the location and size of the control point–specific ROIs, an analysis of the repositioning capabilities of various apertures was assessed as follows. For each BEV, 3000 rectangular apertures shaped by the multileaf collimators (MLCs) were generated with a randomly sampled central position and size ranging from 0.375 to 37.5 cm^2^. The size and position of each aperture was used to create two cropped images of the DRRs; one of the images was shifted laterally and/or vertically with 100 simulated shifts on a grid (up to ±2 mm in steps of 0.04 mm in each direction perpendicular to the BEV at isocenter). For each shift, the shifted image (shifted anatomy) was registered to the unshifted imaged (unshifted anatomy) with the Mattes mutual information in MATLAB. For each BEV, the top 10% of apertures that minimized the mean and standard deviation of registration errors from the simulated shifts (on a grid) were selected for contribution to a composite image. Binary masks of these apertures were overlaid and summed (i.e., each time a voxel of the unshifted DRR was included in a binary mask, the value of that pixel was increased by 1) to create topographical maps, which highlighted anatomy that was commonly included in the best‐performing apertures; an example of one map is shown in Figure 3a as an overlay on top of the DRR with the BB present for illustrative purposes (it was removed for DRR image registration purposes as described previously). Imaging plans comprising control point–specific apertures were created based on discrete threshold levels from the topographical maps shown in Figure 3b. Higher thresholds represented smaller apertures. The apertures were defined by the bounding rectangle for a given isovalue line and made as rectangles with the MLC and the jaws shown in Figure 3c. The impact of threshold‐level on the mean registration error was assessed for known shifts applied during an imaging arc.

To assess the feasibility of the thresholded imaging plans derived from the 3D‐printed skull analysis for use with different clinical cases, an imaging plan (with the 60% thresholded aperture) was applied to DRRs generated for six anonymized, previously treated clinical SRS patients with skull volumes ranging from 2.5 × 10^3^ to 3.5 × 10^3^ cm^3^. To scale the location and size of the ROI apertures, we calculated the cubed root of the relative volumetric scaling factor between the case in question and the 3D‐printed skull for which the ROI apertures were derived; this factor was used to scale each dimension of the aperture (*X*/*Y* extent) as well as the vector position of the aperture with respect to isocenter (location of the metal‐BB) for each BEV. For each case, the grid‐shifting analysis described earlier for deriving the apertures was applied for each BEV to assess the mean registration error within a ±2.5 mm range.

In an effort to quantitatively characterize image information that yields sub‐mm registration results, two characteristics of the anatomy seen within the ROIs (delineated by the apertures created in the analysis earlier, and restricting the analysis to apertures greater or equal to 4 cm^2^) were investigated. For the purposes of this analysis, we have separated apertures into two groups: “Good” apertures were those that produced a range of registration errors (defined by difference between the 95th percentile and 5th percentile errors) less than 0.1 mm, and a mean error less than ±0.1 mm across the entire shift grid. “Bad” apertures were defined as ones that possessed a range of errors greater than 1 mm.

First, the 2D‐directional gradient of the image contained in each aperture was calculated. The angular directions of the vectors that comprised the gradient images were binned into a histogram with eight bins, each having a 45 degree directional span. The contribution of each pixel was weighted by the magnitude of the gradient in that pixel. For the purpose of evaluating the characteristics of good versus bad imaging apertures, the polar histograms were parameterized by the variance of counts across the apertures and normalized by the sum of counts in the histogram.

Second, the mutual information (MI) between the anatomy contained within a given aperture and the same anatomy when it is shifted was calculated (using a grid‐based shifting pattern as described earlier). This produced a 2D array of 121 MI values with the central element having the highest value (i.e., unshifted images have the highest possible MI score) and values decrease for all shifted positions. This information was condensed by calculating the mean difference among all pixels with respect to the central pixel.

The condensed information for both the gradient polar histograms and MI mean difference were calculated for 30 good and 30 bad apertures at gantry angles ranging from 0 to 360 degree in 45 degree increments (i.e., a total of 480 apertures). Scatter plots of the parameterized polar plots against the parameterized MI data were generated to determine if either the metric was predictive of aperture quality.

### 2.4 Motion correction and targeting accuracy

Two forms of positional errors were explored in this investigation, namely, (1) mechanical imperfections of the dynamic motions of the linac (gantry rotation at different couch positions) during delivery and (2) simulated Intrafractional motion. In the following subsections, the methodology for detecting and correcting for motions with the tracking of a high‐*Z* fiducial, as well as anatomical image registration, is explained. In addition, the simulation of intrafractional motion, and the comparison of the performance of the two repositioning strategies is described.

#### 2.4.1 Repositioning by tracking high‐*Z* fiducial

**FIGURE 4:**
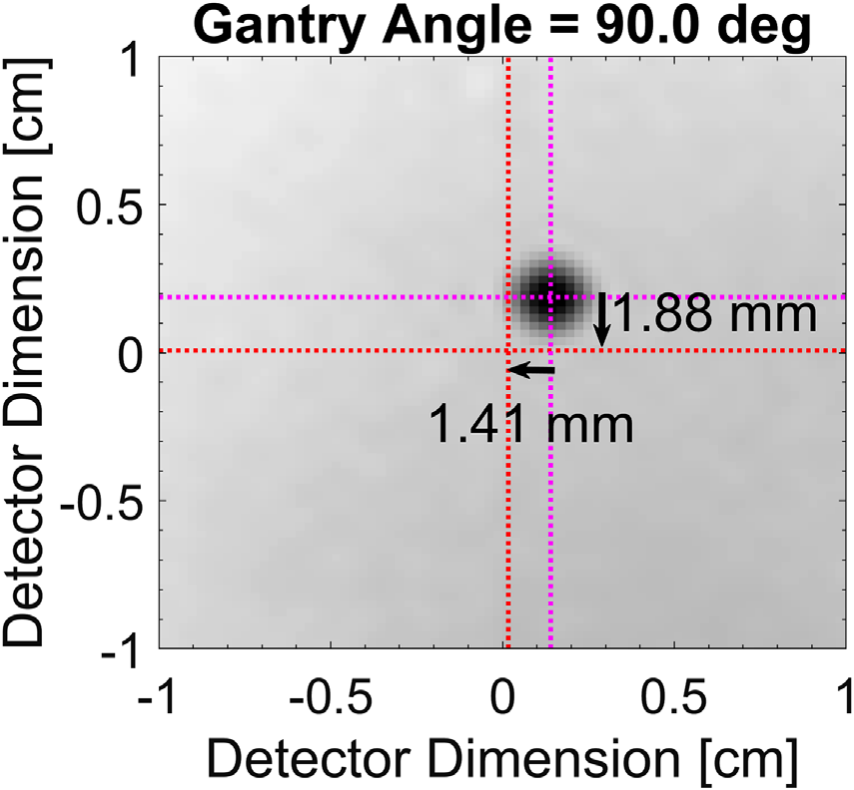
Example of the detection of the center of the BB (magenta line intersection) with respect to the center of the electronic portal imaging device (red line intersection). The detected shifts for this control point are shown for the case of a sudden 1.5 mm shift along each linear couch axis with the gantry at 90 degrees and the couch at 0 degrees.

Following the method described in Parsons et al.,^24^ a small aperture was created with the jaws (2.5 × 2.5 cm^2^) for imaging. The center of the metal‐BB was identified using a maximum convolution approach that maximized values within the BB and zeroed all objects that appeared larger than the physical size of the BB. The center of the BB was then compared with the center of the EPID as depicted in Figure 4. The deviation of the BB was used to derive the necessary couch motions to position the BB at the center of the EPID with

$$\left[\begin{array}{c} \Delta Lat_{\theta ,\varphi }\\ \Delta Lng_{\theta ,\varphi }\\ \Delta Vrt_{\theta ,\varphi } \end{array}\right]=\left[\begin{array}{ccc} \cos\varphi & -\sin\varphi & 0\\ \sin\varphi & \cos\varphi & 0\\ 0 & 0 & 1 \end{array}\right]\;\;\left[\begin{array}{c} \left(BB_{x}-EPID_{x}\right)\cos\vartheta \\ \left(BB_{y}-EPID_{y}\right)\\ \left(BB_{x}-EPID_{x}\right)\sin\vartheta \end{array}\right]$$

where *BB_x_*_/_ *_y_* is the detected center of the BB, and *EPID_x_*_/_ *_y_* is the center of the EPID in the *x*‐ and *y*‐direction for a given BEV, respectively. The angles *θ* and *ϕ* are the gantry and couch angle, respectively.

#### 2.4.2 Repositiong with Anatomical Image Registration

**FIGURE 5:**
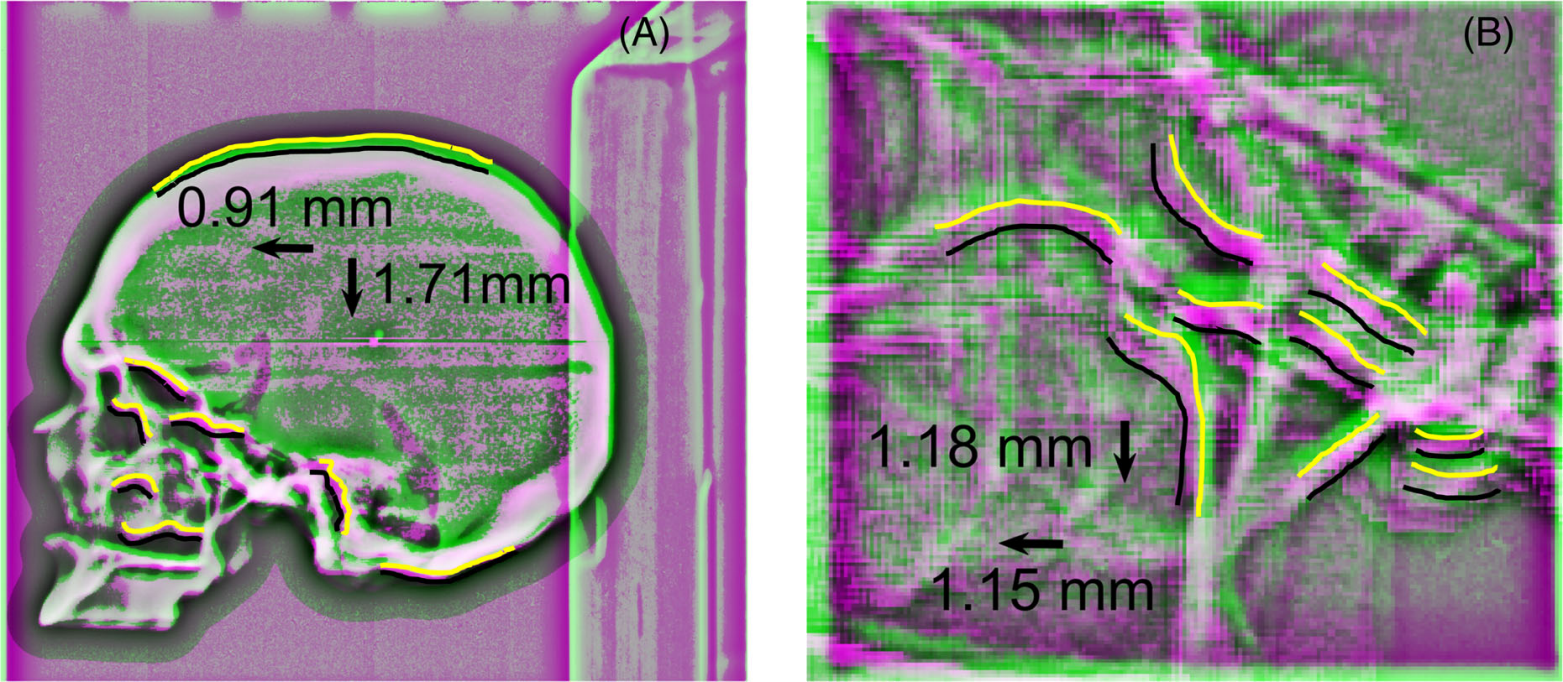
Example of anatomical registration between megavoltage (MV) image with the electronic portal imaging device (EPID) (green overlay) and a MATLAB‐generated digitally reconstructed radiograph (DRR) (magenta overlay). Yellow lines were drawn to highlight example edges of anatomical features in the EPID image, whereas black lines depict the same example edges in the DRR: (a) registration with an open field (22 × 22 cm^2^); (b) registration with an 80% thresholded aperture described in Figure 3. The detected shifts for this control point are shown for the case of a sudden 1.5 mm shift along each linear couch axis with the gantry at 90 degrees and the couch at 0 degrees.

To perform anatomy‐based registration, images were preprocessed with normalization and histogram equalization.^25^ Following preprocessing, images were registered using the imregister function in MATLAB with the Mattes MI using all pixels for registration, and an initial radius of 6.25 × 10^−4^. The deviation of the EPID image from the DRR (shown with the BB present for illustrative purposes) as depicted in Figure 5 was used to calculate the necessary couch motions to align the phantom with respect to the BEV as described in Section 2.4.1 earlier. Two different forms of anatomical imaging were acquired: (1) utilizing open‐field anatomical imaging (shown in Figure 5a) with a 22 × 22 cm^2^ field, which will herein be referred to as ANA_Open_ and (2) utilizing control point–specific apertures as depicted in Figure 5b, which will herein be referred to as ANA_60_ or ANA_80_ for the 60% and 80% thresholded apertures, respectively. With the ROI imaging, the EPID images were cropped to the physical size of the aperture prior to registration. None of the ROI apertures considered in the work imaged the BB embedded in the center of the skull. The detected couch motions for repositioning with anatomical registration was compared to the same corrections derived with the tracking of the metal‐BB; the tracking of the metal‐BB was considered the gold standard for repositioning due to the high‐contrast presence for any BEV.

#### 2.4.3 Simulated Intrafractional Motion

In this study, intrafractional motion was simulated with simplistic motion traces to evaluate the repositioning capabilities of the registration algorithm when there were known deviations of the phantom. For one motion trace, the phantom was linearly moved 1.5 mm in each direction over the full treatment duration (i.e., first control point had 0 mm of motion; in the last control point, the couch was shifted 1.5 mm in each linear couch axis). The other motion trace emulated a sudden 1.5 mm shift in each linear couch axis that occurred halfway through the axial arc (the first arc delivered). For both cases (motion or no motion), positioning errors were assessed by comparing the results of image registration (between DRR and EPID) to the tracking of a metal‐BB with respect to the center the EPID. This ensured that any positioning errors that resulted from mechanical issues like EPID sag did not impact the comparison of the two registration techniques.

### 2.5 Imaging dose calculation

Dose distributions for each imaging arc with a given aperture design was calculated using EGSnrc. The treatment head of the TrueBeam STx platform was simulated using a previously validated 2.5 MV photon beam generated in VirtuaLinac (Varian Medical Systems, Inc., Palo Alto, CA).^26^ The phase space was located 73 cm above isocenter and was validated as accurate to better than 2% compared to measured depth–dose and off‐axis profiles. This served as the input for a BEAMnrc model containing the jaws,^27^ HDMLC, and Mylar exit window. These three components were modeled using exact geometric and material specifications provided by Varian Medical Systems. This was used as an input to DOSXYZnrc.^28^ The phantom was created with voxel sizes of 9.62 mm^3^ from the CT of the ATOM (Model 701, Computerized Imaging Reference Systems, Inc. Norfolk, VA) head phantom. An ECUT = 0.512 MeV and a PCUT = 0.010 MeV were used with 10^10^ histories for each arc. An equal number of monitor units was delivered for each imaging control point using the definition of monitor units defined for Source‐21 within DOSXYZnrc.

## 3 RESULTS AND DISCUSSION

### 3.1 Characterization of high‐quality imaging apertures

**FIGURE 6:**
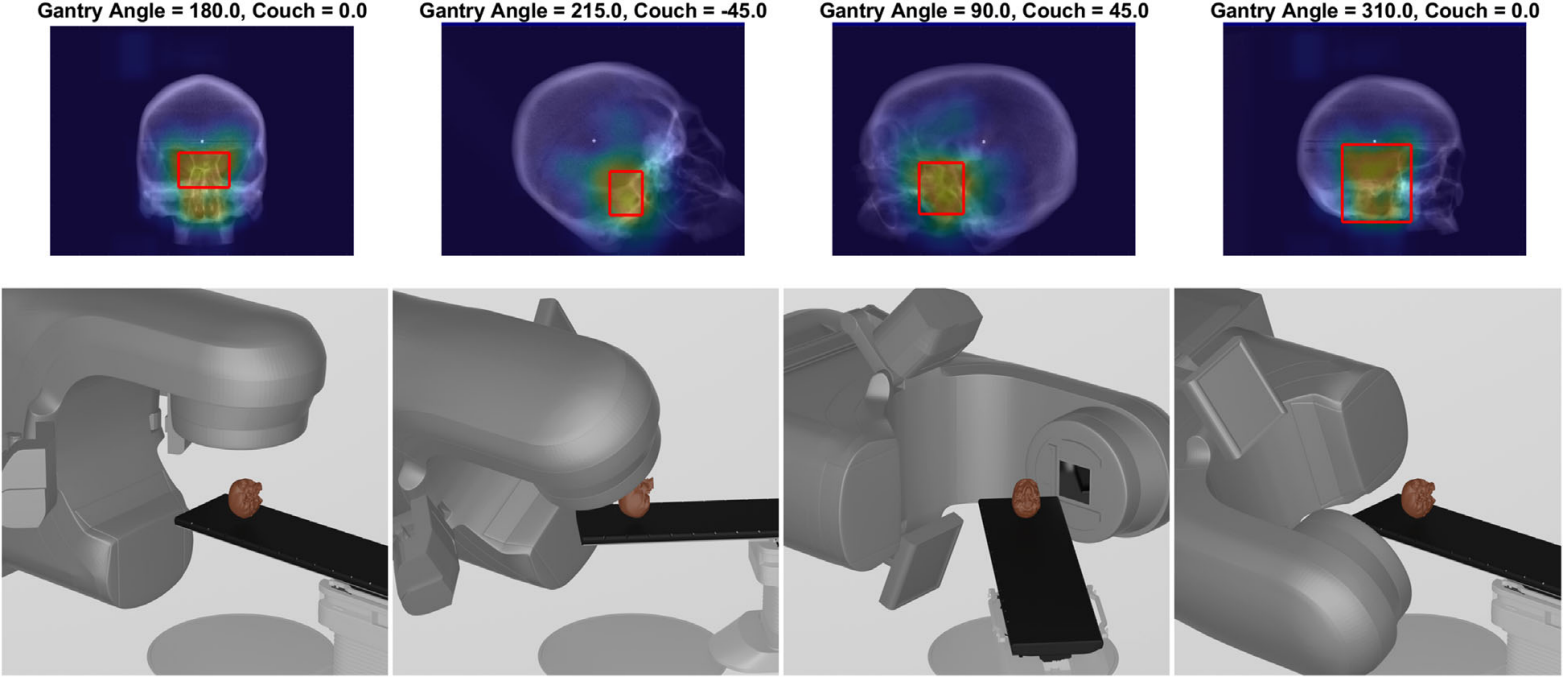
Examples of the top 10% of apertures that minimized registrations errors for four different beam's eye view (BEV) depicted as a color wash overlaid onto a digitally reconstructed radiograph (DRR). Bright colors highlight the anatomical regions shared by the proportion of the top 10% of apertures. The red box articulates the 80% threshold level for the creation of an imaging aperture for the respective BEV. The accompanying couch and gantry positions are depicted in the row below the BEV.

A few examples of the outcomes from the aperture‐searching algorithm described in Section 2.2 are shown in Figure 6. Here, a topographical map, as depicted in Figure 3a,b, is shown as a color wash over a DRR for a given BEV, with a bounding rectangle delineating a ROI for imaging with the 80% threshold line. The accompanying gantry and couch orientations for the depicted beams are shown as a rendering of the linac with the phantom included in the row below the color wash. The color wash depicted in these images highlight (as bright colors from the color wash) anatomical features that were common to the top 10% of apertures, which exhibited minimal registration errors. Qualitatively, these examples also depict regions that would be poor for imaging to minimize positional errors (identified as dark colors in the wash). Regions with poor registration capabilities tend to contain a lack of bony landmarks (such as the middle of the skull). Potentially counter intuitively, some regions of bony anatomy do not feature prominently in the highlighted apertures. This meant that those regions did not meet the criteria for “good” apertures described previously and suggests that those regions may estimate registration‐derived shifts that are erroneous in a subset of motion cases (i.e., they could have excellent registration results, but only in a subset of patient motion directions).

**FIGURE 7:**
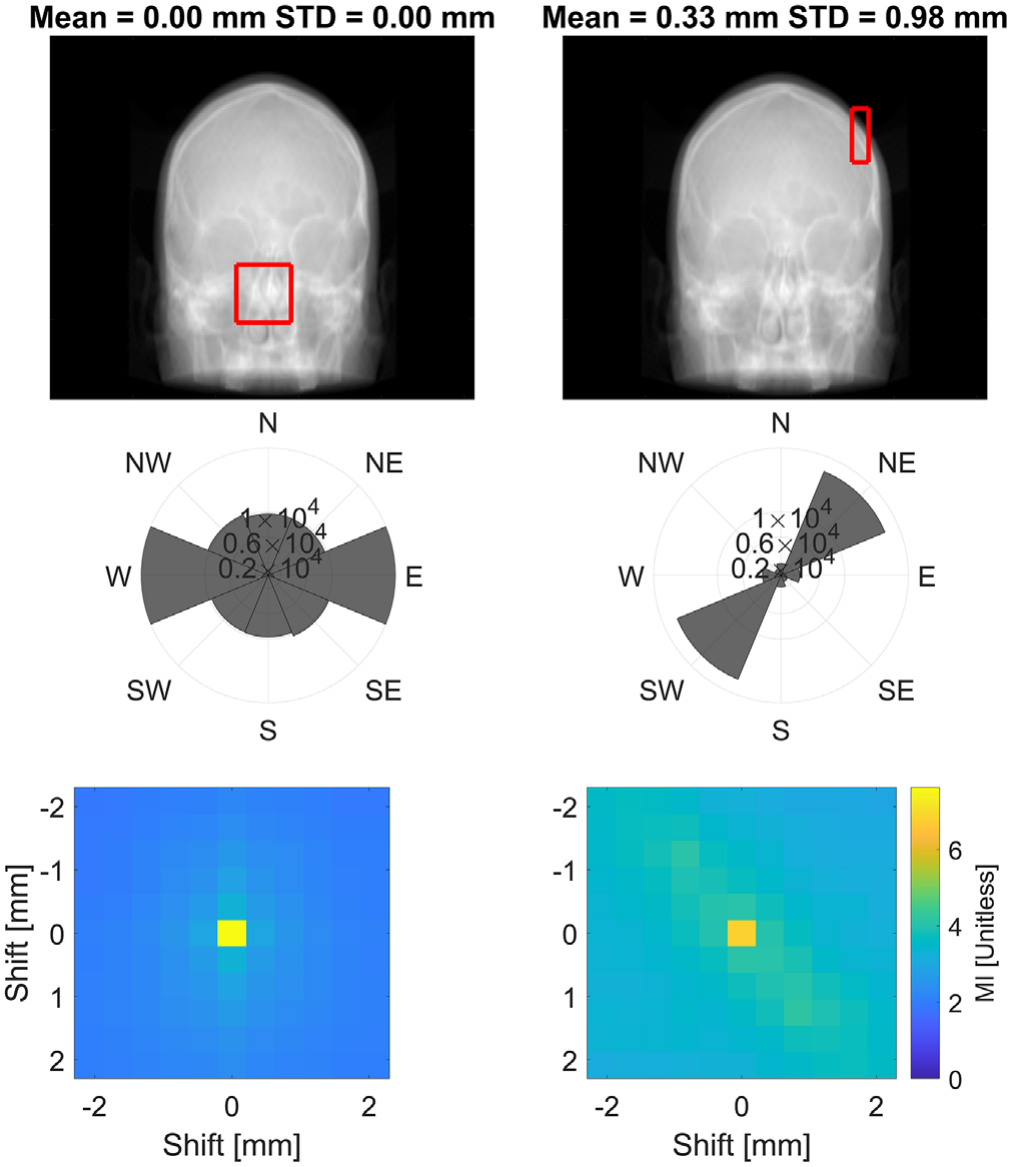
The second row depicts a polar histogram where the counts are the pixel values of weighted‐gradient image of the anatomy contained within the aperture (depicted by red rectangle in the top row). The bottom row depicts the mutual information between unshifted and shifted anatomy contained within the aperture for 2D‐shifts with respect to the beam’ s eye view.

**FIGURE 8:**
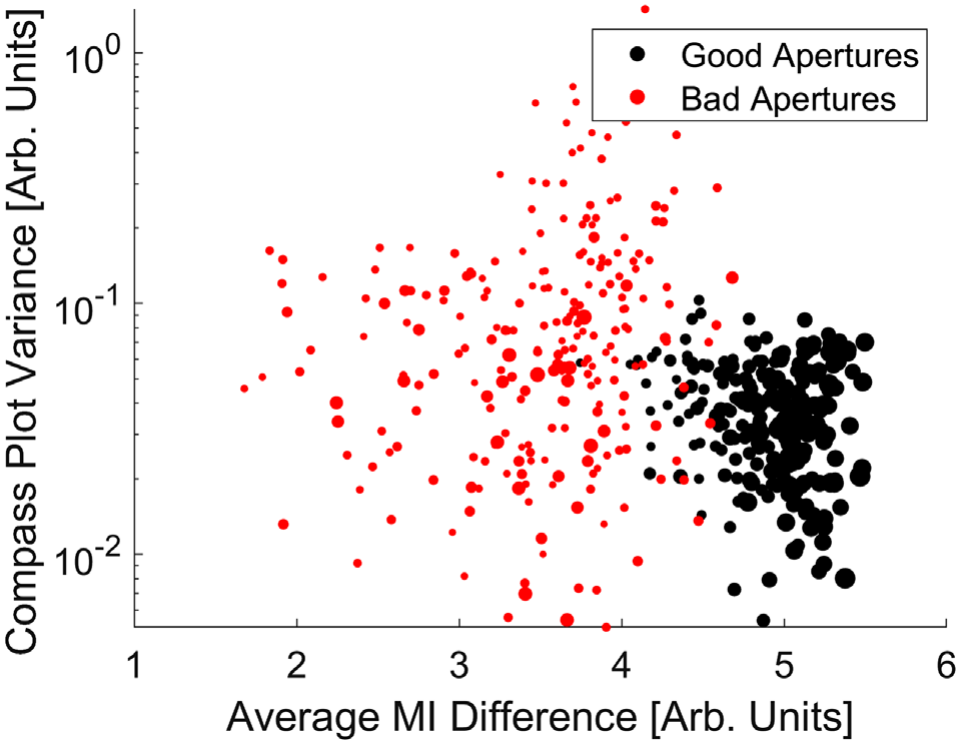
Variance of compass plot depicted in Figure 7 normalized by the total counts with respect to the average mutual information (MI) difference derived from the bottom row plots in Figure 7. Black circles represent good apertures with a range of registration errors less than 0.1 mm and a mean registration error less than 0.1 mm, and red circles represent bad apertures with a range of registration errors greater than 1 mm. The sizes of the markers are indicative of the size of the aperture. The smallest markers represent at 4 cm^2^ field size, and the largest markers represent a 36 cm^2^ field size.

An example of a good and bad aperture for repositioning is demonstrated in Figure 7. Here the polar histogram for the good aperture is shown to contain a more uniform distribution of directional information, whereas the bad aperture is heavily unidirectional. Additionally, the MI distribution for simulated shifts depicts a rapid drop‐off in all directions with the good aperture, whereas the bad aperture depicts a sharper drop‐off in some directions and not in others (implying repositioning capabilities would be strong in some directions and weaker in others); moreover, the magnitude of the central pixel is lower with the bad aperture, which implies a smaller quantity of high‐entropy content. As described in Section 2.3, the behavior of these data across multiple apertures and BEVs was condensed and is depicted in Figure 8. Within this parameterized data, the average polar histogram of good apertures contain 112% more counts than bad apertures. This could be the result of apertures being larger and/or containing a relatively larger presence of high‐contrast feature with strong directional gradients (e.g., bone bordering soft tissue). There is an evident clustering of data between the two aperture subsets. Although the magnitude of variance of the polar histogram alone does not appear to be enough to distinguish between good and bad apertures, the average MI difference does as it is 40% larger for good apertures when compared with bad apertures. The mean and standard deviation of the compass plot variance is 0.11 ± 0.15 and 0.04 ± 0.02 for bad and good apertures, respectively. The variance metric in conjunction with the average MI difference could be used to identify apertures that have strong repositioning capabilities but only in a few directions.

### 3.2 Dose reduction with ROI apertures

**FIGURE 9:**
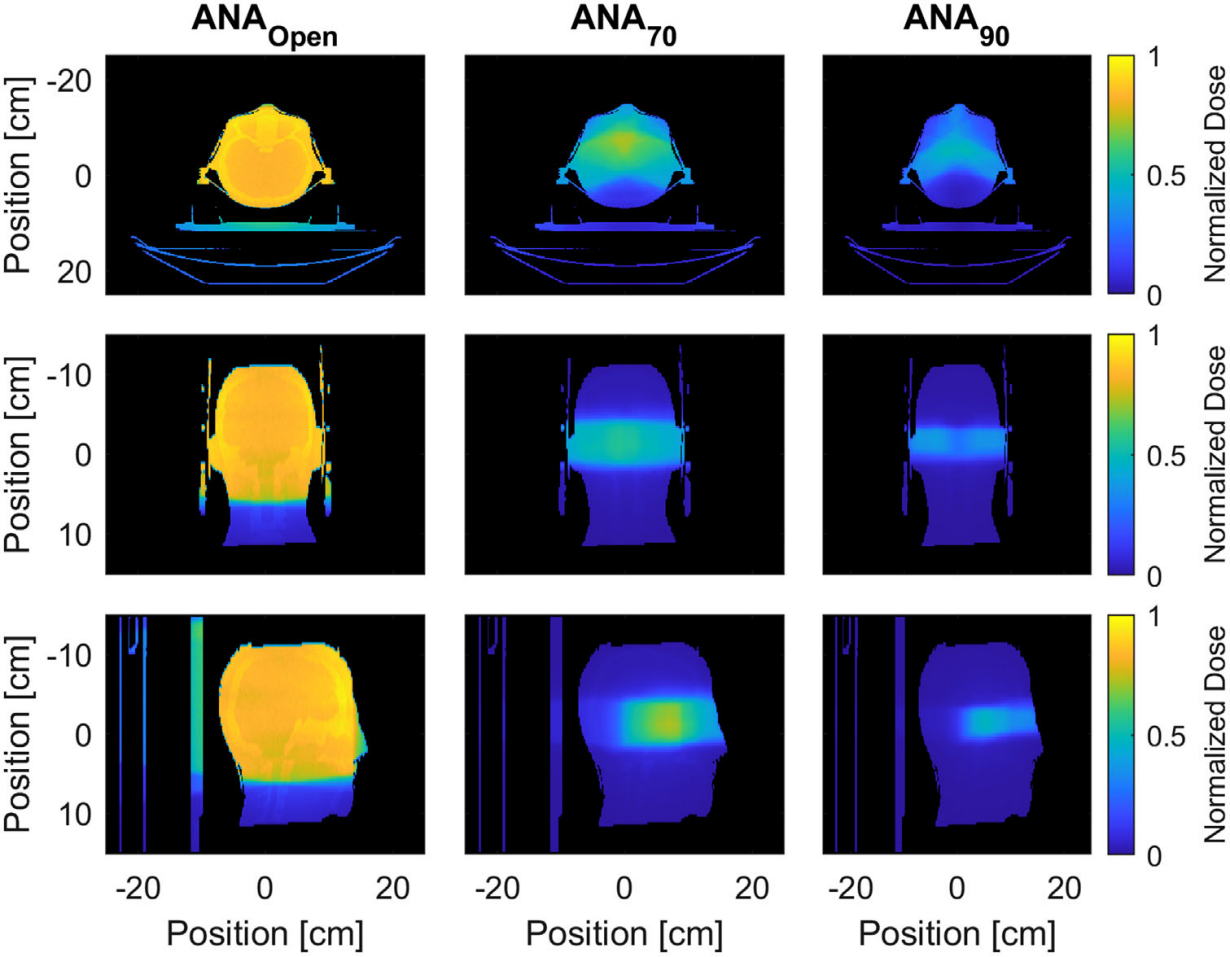
Dose washes for the central axial, coronal, and sagittal planes calculated with Monte Carlo for ANA_Open_ (22 × 22 cm^2^ field size), ANA_70_ (70% thresholded aperture design), and ANA_90_ (90% thresholded aperture design). Each dose wash was normalized to the max dose of the ANA_Open_ plan.

Reducing of the size of the imaging aperture leads to a reduction in the imaging dose. There was an 83% reduction in the dose received by 50% of the volume (*D*_50_) throughout the skull for an axial imaging arc with the 50% thresholded plan when compared to imaging plan with an open aperture (22 × 22 cm^2^). From the 50% to 90% thresholded imaging plan, the *D*_−50_ throughout the skull was reduced by 87%. The normalized integral dose with decreasing field sizes (notated by threshold levels from the surface plot in Figure 3) decreased in a linear fashion from the 50% to 90% threshold. Qualitatively these results are visualized with a dose wash calculated with MC simulations in Figure 9, where this is a notable reduction in the magnitude and size of the imaging dose distribution. The imaging arc simulations with a 22 × 22 cm^2^ field had a less than 4% and 3% voxel uncertainty for doses greater than or equal to 50% and 90% of the maximum dose, respectively.

### 3.3 Registration errors

Pre‐Imaging alignment with a CBCT reported positional differences with the planning CT of less than 0.1 mm in any dimension as well as less than 0.1 degrees of couch rotation (yaw). The maximum detected offset of the BB from isocenter without simulated intrafractional motion is shown in Table 2. The errors represented in this table would be indicative of a single point for calculating the isocentric sphere comparably calculated with a Winston–Lutz test.

**TABLE 2:** Maximum detected offsets in the lateral, vertical, and longitudinal couch motions (in mm) of the metal‐BB when the couch is positioned at idealized isocenter

Couch angle	−45	0	45
Lateral	0.71	0.15	0.10
Vertical	0.18	0.23	0.18
Longitudinal	0.42	0.34	0.18

**FIGURE 10:**
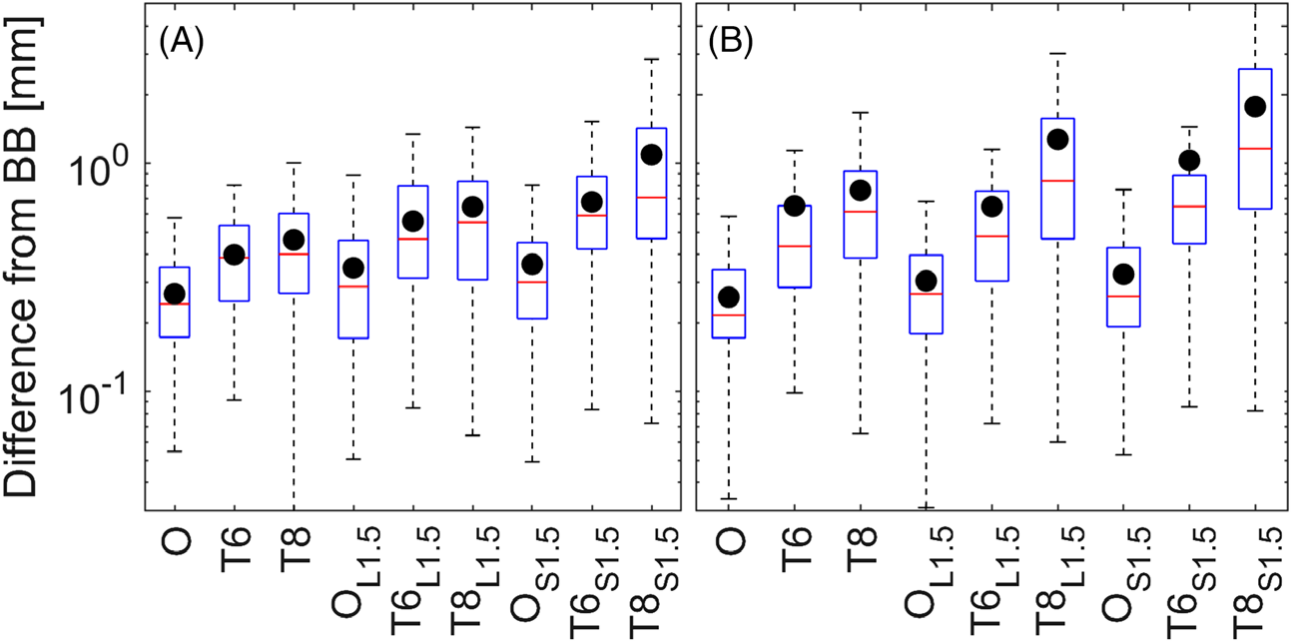
Registration errors for the various imaging acquisitions with respect to BB‐tracking: Part (a) represents 3D‐errors detected using image registration with respect to BB‐tracking; part (b) represents 2D‐errors with respect to BB‐tracking for each beam's eye view. The mean value is depicted by the black circles overlaid on each box. L1.5, linear shifting up to 1.5 mm while imaging; O, open field imaging; S1.5, sudden shift of 1.5 mm while imaging; T6, thresholded imaging at the 60% isoline level; T8, thresholded imaging at the 80% isoline level

During simulated linear motion, the 2D positional errors (i.e., positional error in the BEV) with respect to BB‐tracking were less than 1 mm in 100%, 89%, and 57% of imaging control points for open‐field imaging (ANA_open_), and thresholded imaging (ANA_60_ and ANA_80_), respectively. During a simulated sudden persistent‐motion, the 2D positional errors with respect to BB‐tracking were less than 1 mm in 100%, 82%, and 42% of imaging control points for open‐field imaging (ANA_open_) and thresholded imaging (ANA_60_ and ANA_80_), respectively. In Figure 10, the repositioning capabilities of imaging with progressively smaller apertures (from an open field with a 22 × 22 cm^2^ aperture to the 80% thresholded plan derived from Figure 3) are presented as the difference from the detected shifts with metal‐BB tracking. In Figure 10a, the 3D repositioning errors based upon the detected couch shifts are shown. All median 3D‐errors are sub‐mm indicated by the centerline in the box. The 2D repositioning errors with respect to the BEV are shown in Figure 10b. All median 2D‐errors are sub‐mm with the exception of the T8 imaging (equivalently ANA_80_ with an 80% thresholded plan but changed for display purposes) with a sudden shift for which a 1.16 mm discrepancy was observed.

**FIGURE 11:**
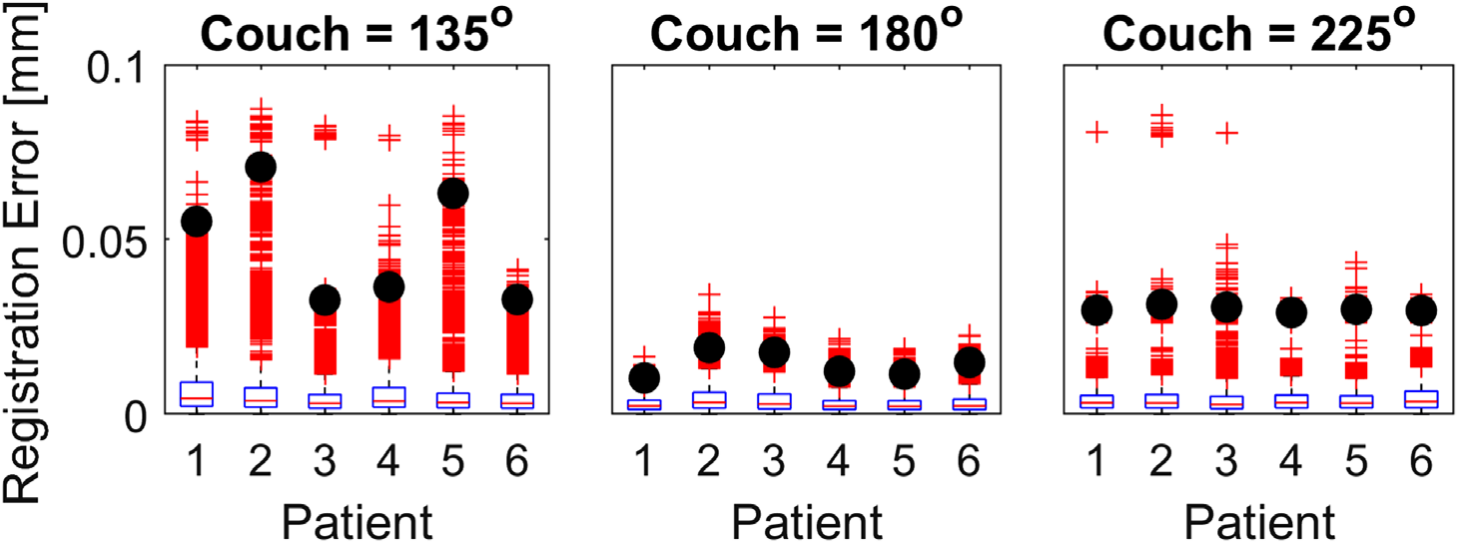
Registration errors detected across all gantry angles (179.9–355 degrees for couch = 135 degrees, 180.1–5 degrees for couch = 225 degrees, and 180.1–175 degrees for couch = 180 degrees; IEC coordinates) and all simulated shifts on a ±2.5 mm grid with respect to the beam's eye view for six clinical cases. Red plus signs indicate outliers that represent the top 25 percentile of registrations errors detected, and the black circles represent the 99th percentile registration error detected.

The registration error detected for the grid‐shifting analysis with the clinical cases using the 60% thresholded aperture imaging plan is shown in Figure 11. For the clinical cases, the 99th percentile of registration errors was less than 0.1 mm across all gantry angles and all simulated shifts on a grid. For patients 1–6, the volumetric scaling factor was determined to be 1.13, 1.18, 1.06, 1.15, 1.16, and 1.06, respectively.

## 4 DISCUSSION

This study investigates the complex relationship that exists between imaging aperture size and location, patient dose and image registration accuracy. Larger aperture plans (ANA_60_) were shown to maintain sub‐mm registration errors, whereas smaller apertures were not always able to do so (ANA_80_). However, given the fact the imaging phantom utilized in this study represented the lower end of the HU range encountered in the skull, clinical images of a real skull may provide improved contrast that could improve registration results, particularly for smaller ROI apertures. Several studies have identified the dose‐reduction possibilities of volume‐of‐interest (VOI) kV‐CBCT,^20^ MV‐CBCT. Robar et al. demonstrated a 39% reduction in dose when reducing the field size from 25 × 25 cm^2^ to a 4 × 4 cm^2^ within a VOI when imaging with a 2.35 MV beam generated with a carbon target.^21^, ^22^ The findings of maximum dose reduction seen within this study were comparable, with a 34% dose reduction with a 2.5 MV beam when comparing a 22 × 22 cm^2^ imaging plan with a 70% thresholded aperture plan with an average aperture size of 19.6 cm^2^. Dose reduction when using a thresholded plan varies on a control point–specific bases due to the varying size of the aperture for each respective BEV. Ding and Munro showed that the *D*_50_ is ∼0.8 cGy for a single 40 × 40 cm^2^ image.^18^ Extrapolating the average dose‐reduction possibilities presented from the Monte Carlo analysis in this work, using an ANA_60_ image would incur ∼0.07 cGy *D*_50_ for the entire skull, and, therefore, ∼10.10 cGy for a full imaging plan (images every 5 degrees, 72 control points for axial arc, and 38 controls per noncoplanar arc). However, this imaging dose is small compared to therapeutic dose in typical cranial SRS plans, which would be 1500–2400 cGy for brain metastases,^29^ or 4778–8500 cGy for trigeminal neuralgia^30^; sensitive structures with strict dose tolerances may necessitate a reduction in imaging frequency. In a clinical setting, this could be realized by imaging at a reduced frequency (i.e., every 20 degrees, or image every 500 MU, which would force more control points in sub‐arcs with high dose delivery). An additional dose‐reduction strategy could also include the reduction of monitor units delivered per image, though this would be accompanied by an increase in image noise. An investigation by Borsavage and colleagues found a 10%–18% reduction in CNR between cortical bone and soft tissue when reducing the imaging dose from two to one cGy with a 2.5 MV beam.^31^

The use of a single planar 2D image for 3D repositioning has the potential to incur positional errors along beamline due to the nature of the trigonometric approach for calculating couch positions from detected 2D shifts. It has been shown in several works that the majority of motions within a thermoplastic mask do not exceed 2.5 mm along any given dimension.^6^, ^15^, ^32^, ^33^, ^34^ Considering extreme positional errors of 3 mm along beamline would only result in dose errors of ∼0.60% based on inverse square changes. The main motivation for implementing the methodology proposed in this work is to minimize dosimetric errors that can occur in‐plane for a given BEV. As shown in previous work in the context of treating with virtual cones, a 1.0 mm linear drift of a 4 mm target along each direction can lead to an 11% reduction of the volume receiving the prescription dose, and a 39% increase in the dose received by the healthy tissue immediately adjacent to the target.^8^ A more complex 2D–3D registration could be utilized with MV/kV imaging as was proposed by Fu and Kuduvalli to better account for beam direction position error.^35^ When utilizing this method to image a head‐and‐neck phantom in 49 different simulated positions (within ±20 mm and ±5 degrees along each orthogonal dimension), they found a mean 3D‐registration error of 0.33 mm. Although a Winston–Lutz test on a linac is usually implemented to validate a sub‐mm isocenter volume,^36^ the reported value is always smaller than the maximum deviation encountered in the analysis, and it only represents a very small sampling of a very large parameter space (i.e., all possible couch/gantry combinations).

Rotational errors were not explicitly addressed in the methodology proposed in this work as separating translation and/or rotational components from single planar images is all but impossible, except for limiting cases where rotations are orthogonal to the imaging plane (e.g., perceived yaw‐rotations for an anterior–posterior MV image). Even with 2D–3D imaging, there exists a complex coupling of translations and rotations, which was explored for 2D–3D MV imaging by Jans et al.^25^ The methodology presented in this work may not be well suited to accurately quantify complex rotational motions; however, it is well suited for identifying discrepancies between BEV DRRs and intra‐treatment images. As such, in addition to permitting the online correction of small positional errors, this methodology could also be utilized to detect positional discrepancies that exceed a user‐defined threshold and trigger a treatment interruption to permit a more robust patient repositioning procedure (e.g., CBCT or stereoscopic imaging).

A method such as the one outlined here has the potential to correct for positional errors that can go otherwise undetected during treatment delivery. However, for large couch angles, imaging would be impossible due to the collision of the gantry with the couch and/or patient. For example, when imaging a head and thoracic cavity phantom with the couch at 90 degrees, we found that imaging was possible for gantry angles from 180 to 200 degrees and 340 to 15 degrees (Varian IEC coordinates). Safe operation of a linac necessitates the avoidance of a collision between the patient and the linac during the motion of its mechanical axes, and, thus, these regions should be quantified. Northway et al. explored this concept by mapping patient‐specific image sets to a library of body contours that were placed on the bed of a virtual linac model. With this model, collisions zones were mapped out for noncoplanar stereotactic body radiation therapy.^37^ This approached could be extended to include a model of the imaging panel. Treatment plans could then be designed to avoid these regions, or at least inform the treatment team of the control points at which position verification imaging would not be possible. This approached could be extended to include a model of the imaging panel. Implementing an imaging protocol as depicted in this investigation would benefit significantly from an efficiency point of view from a fast‐switching target similar to the work explored by Berbeco et al.^38^ and Yewondwossen et al.^39^

## 5 CONCLUSIONS

This investigation has demonstrated the capability of ROI control point–specific MV imaging to detect and correct for intrafraction motion observed during cranial SRS therapies. The use of ROI MV‐imaging has been shown to reduce the accrued imaging dose while balancing the proportion of visible anatomy needed to detect and correct for motions observed from a BEV. Although the method was based on a single skull, we have demonstrated that the data are generalizable. Compared to an open field, aperture‐specific corrections can reduce imaging dose by up to 83% with a three‐arc plan. For a 1.5 mm linear drift in phantom motion along each direction, 60% threshold design demonstrated a mean registration error of 0.56 ± 0.33 mm. This is the first work that we are aware of to present a method for the construction of small, robust imaging apertures that balances the objectives of accurate intrafraction position detection and dose minimization.

## FINANCIAL DISCLOSURES

CC, DP, and AS have no financial disclosures.

## CONFLICT OF INTEREST

The authors have no conflicts of interest to disclose.

## AUTHOR CONTRIBUTION

Cody Church was responsible for study design, data acquisition and analysis, and manuscript writing. David Parsons was responsible for study design, evaluation of analyzed data, and revision of the manuscript. Alasdair Syme was responsible for study design, evaluation of analyzed data, and revision of the manuscript.

## Data Availability

The data that support the findings of this study are available from the corresponding author upon reasonable request.
